# Exploration of Intersections and Divergences of Long COVID and Chronic Fatigue Syndrome

**DOI:** 10.7759/cureus.90607

**Published:** 2025-08-20

**Authors:** Joao A Kouyoumdjian, Leticia A Yamamoto, Carla R Graca

**Affiliations:** 1 Neurological Sciences, Psychiatry and Medical Psychology, Faculty of Medicine of Sao Jose do Rio Preto, Sao Paulo, BRA

**Keywords:** chronic fatigue syndrome, fatigue, long covid, myalgic encephalomyelitis, sars-cov-2

## Abstract

Background: Fatigue is the most common symptom of Long COVID (LC), defined by persistent or newly emerging symptoms that develop at least three months after an initial SARS-CoV-2 infection, in the absence of other identifiable cause. This study investigates the prevalence of myalgic encephalomyelitis/chronic fatigue syndrome (ME/CFS) as a potential comorbidity of LC.

Methods: The study enrolled 37 adult controls with no documented SARS-CoV-2 infection and 32 individuals with a history of infection, categorized as LC-yes (with LC symptoms) and LC-no (without LC symptoms). ME/CFS diagnosis was based on the International Consensus Criteria (ICC).

Results: Among LC-yes cases, the most frequently reported symptoms included post-exertional malaise (PEM); neurosensory, perceptual, or motor disturbances; cognitive impairment; sleep disturbances; pain; impaired thermoregulation; and flu-like symptoms, all occurring significantly more than in the LC-no or control groups. All individuals in the LC-yes group reported PEM. ME/CFS was diagnosed in three LC-yes cases (18.8%), one LC-no case (6.7%), and four control subjects (10.8%), with no statistically significant differences observed among groups. Experiencing more than six symptoms during acute infection, such as fatigue, loss of taste or smell, headache, fever, cough, myalgia, sore throat, shortness of breath, rhinorrhea, and diarrhea, was associated with a twofold higher risk of developing LC.

Conclusion: A substantial proportion of LC-yes individuals experienced PEM; neurosensory, perceptual, or motor disturbances; cognitive impairment; and sleep disturbances, with rates significantly exceeding those in the LC-no and control groups. Nevertheless, only a minority of LC-yes cases (18.8%) satisfied criteria for the ME/CFS, and the prevalence did not significantly differ from LC-no and controls. These findings suggest that while many symptoms of LC overlap with those of ME/CFS, only a subset of LC cases meet established ME/CFS diagnostic criteria.

## Introduction

In December 2019, Wuhan, China, experienced a surge in pneumonia cases that rapidly spread worldwide. Given their clinical similarity to cases of severe acute respiratory syndrome (SARS), caused by the 2003 SARS-CoV-1 virus, these pneumonia cases were quickly linked to a novel coronavirus, later named SARS-CoV-2 [1]. Since then, about 778 million cases and more than 7 million deaths from COVID-19 have been reported globally [2]. The World Health Organization (WHO) defines long COVID (LC), also referred to as post-COVID-19 condition, as the persistence or emergence of new symptoms beyond three months after the initial SARS-CoV-2 infection, with no alternative diagnosis [3]. This definition highlights the diverse and often disabling nature of the LC, which has now been recognized as a significant global health concern [4].

In the United States, survey data show that about 7% of adults have experienced LC, and it has affected millions of others worldwide [5]. Initially, symptoms such as cognitive impairment, neuromuscular issues, depression, and fatigue were linked to post-intensive care syndrome. However, this idea was challenged when similar symptoms appeared in tens of thousands of patients from the pandemic's first wave, many of whom had mild symptoms and never needed hospitalization [5].

LC is a multifactorial condition characterized by severe new or persistent symptoms, including post-exertional malaise (PEM), fatigue, and cognitive impairment (commonly referred to as "brain fog"). The pathological features of LC involve dysregulation across multiple systems - immune, cardiovascular, metabolic, gastrointestinal, nervous, and autonomic - that substantially overlap with those observed in myalgic encephalomyelitis/chronic fatigue syndrome (ME/CFS). Current evidence suggests that a significant proportion of LC patients may ultimately meet diagnostic criteria for ME/CFS.

ME/CFS is predominantly characterized by PEM, profound severe fatigue, cognitive dysfunction, unrefreshing sleep, myalgia, polyarthralgia, headache, and autonomic disturbances, each of which can have a substantial impact on daily functioning [6,7]. The potential link between ME/CFS and LC has garnered widespread attention, as not all individuals with LC satisfy the diagnostic criteria for ME/CFS. Notably, symptoms such as PEM, fatigue, and sleep disturbances observed in LC may also result from other viral infections, giving rise to post-viral fatigue syndrome (PVFS). These overlapping clinical features support the hypothesis that LC symptoms may manifest as a distinct condition or, alternatively, fall within the clinical spectrum of PVFS or ME/CFS.

Several diagnostic criteria exist for ME/CFS. The Fukuda criteria, which have been widely utilized for many years, do not capture several common symptoms associated with ME/CFS, limiting their diagnostic sensitivity [8]. In contrast, the more recent International Consensus Criteria (ICC), developed from the Canadian Consensus Criteria, provide a more comprehensive and nuanced approach [6]. The ICC emphasizes the multi-system nature of ME/CFS and addresses prior limitations by excluding the requirement for a minimum symptom duration of six months, thereby enabling earlier diagnosis and more accurately reflecting the disease complexity.

The objective of this study was to apply the ICC criteria to a cohort of individuals without documented SARS-CoV-2 infection and to compare the findings with two groups of SARS-CoV-2-positive subjects: one comprising asymptomatic individuals and the other consisting of patients exhibiting new or persistent symptoms six months post-infection.

## Materials and methods

Study population

Between July 2021 and June 2024, we conducted a random selection and evaluation of adult control subjects and patients of both sexes from our medical school and teaching hospital. Patients with a confirmed history of SARS-CoV-2 infection were stratified into two gender-balanced groups: the LC-no group, comprising asymptomatic individuals, and the LC-yes group, including those with persistent or newly developed symptoms six months following the acute infection. Inclusion criteria required either a symptomatic SARS-CoV-2 infection confirmed by a positive polymerase chain reaction (PCR) test or serological evidence of antibodies. Exclusion criteria encompassed: (a) admission to the ICU, with or without ventilatory support; a 24-hour hospital admission for observation followed by discharge was not an exclusion criterion, (b) pre-existing neuromuscular diseases, (c) antecedent persistent fatigue, and (d) chronic systemic conditions, including diabetes and chronic kidney disease.

Myalgic encephalomyelitis/chronic fatigue syndrome score

ME/CFS was diagnosed based on the ICC if the individual experienced PEM (domain A) and exhibited at least one symptom from each of the domains B, C, and D (Table 1) [6]. The severity of PEM was assessed by scoring the five subcategories as follows: (0) no symptoms, (1) occasional, (2) about half the time, (3) most of the time, and (4) continuous symptoms. The ICC helps identify severe cases of ME/CFS by defining the clinical benchmarks used to evaluate fatigue, sleep disturbances, cognitive impairments, and dysfunctions of the autonomic nervous, neuroendocrine, and immune systems (Table 1) [6].

**Table 1:** The ICC for ME/CFS diagnostic score PEM: Post-exertional malaise; ICC: International Consensus Criteria; ME: Myalgic encephalomyelitis; CFS: Chronic fatigue syndrome

Domain A	PEM and persistent and debilitating fatigue
	a. Minimal exercise with disproportionate tiredness
	b. Exhaustion after light activity
	c. Pain after non-strenuous activities
	d. Feeling "dead tired" after exercise
	e. Mentally tired after a small activity
Domain B	Neurological impairment
	a. Neurocognitive impairments
	b. Pain
	c. Sleep disturbance
	d. Neurosensory, perceptual, and motor disturbances
Domain C	Immune, gastrointestinal, and genitourinary impairments
	a. Flu-like symptoms
	b. Susceptibility to viral infections with prolonged recovery periods
	c. Gastrointestinal tract symptoms
	d. Genitourinary symptoms
	e. Sensitivities to food, medications, odors, or chemicals
Domain D	Energy production and transportation impairments
	a. Cardiovascular symptoms
	b. Respiratory symptoms
	c. Loss of thermoregulatory stability
	d. Intolerance of extreme temperatures

Since this study investigated a medical condition (ME/CFS) that lacks a definitive biological marker, it relied on structured questionnaires based on the ICC [6]. To ensure consistency and accuracy, all interviews were conducted in person by the same researcher. Each interview took place in a comfortable setting, lasted about 30 minutes, and followed a standardized set of questions that were applied uniformly across all participant groups. The interviewer made sure that each participant clearly understood the questions before answering. All participants had an educational level sufficient to understand and accurately respond to the questionnaire.

The diagnostic criteria proposed by Carruthers et al. were used to establish a diagnosis of ME/CFS [6]. However, although Domain A (PEM) is required, it does not have additional subdivisions that could help improve comparisons across different groups. In the original article by Carruthers et al., the suggested subgroup features include: 1) Rapid physical and/or cognitive fatigability from minimal exertion, leading to relapse; 2) Post-exertional symptom exacerbation (e.g., flu-like symptoms, pain, worsening of prior symptoms); 3) Post-exertional exhaustion, immediate or delayed by hours or days; 4) Prolonged recovery (≥24 hours; relapses may last days to weeks or longer); 5) Low stamina, causing reduced pre-illness activity levels [6].

Because fatigue is often an isolated symptom in LC, conducting detailed comparative analyses using the questions from Carruthers et al. would be difficult [6]. To facilitate easier participant responses, we simplified the subdivision of Domain A (PEM) and incorporated a frequency scale as follows: 1) Minimal exertion causing disproportionate tiredness; 2) Exhaustion following light activity; 3) Pain triggered by non-strenuous activities; 4) Feeling profoundly fatigued ("dead tired") after exercise; 5) Experiencing mental exhaustion after minimal activity. Consistent with previous methods, the severity of PEM was also scored across these five subcategories using a frequency scale: 0 (no symptoms), 1 (occasional), 2 (about half the time), 3 (most of the time), and 4 (continuous symptoms).

The diagnostic criteria for ME/CFS remain unchanged; however, the assessment of fatigue has been expanded to incorporate additional subgroups. This approach enabled a more robust and reliable severity across the different participant groups.

Clinical summary

13 symptoms of acute SARS-CoV-2 infection were recorded, including fever, cough, sore throat, runny nose, shortness of breath, phlegm, muscle pain, tiredness, fatigue, headache, diarrhea, and loss of smell and taste.

Tiredness is a nonspecific, subjective sensation characterized by a reduced sense of energy or feeling exhausted. It may arise from a variety of physiological, psychological, or environmental factors. In contrast, fatigue is a more persistent and profound experience of physical, mental, or emotional exhaustion that is disproportionate to recent exertion and is not alleviated by rest. Although tiredness and fatigue may appear similar in clinical history, fatigue is typically indicative of an underlying medical, psychological, or physiological condition. For the purposes of this study, fatigue is considered present when symptoms persist despite adequate rest.

Ethical consideration

The study received ethical approval from the ethics committee of the Faculdade de Medicina de São José do Rio Preto in São Paulo, Brazil, with protocol number 4904517, where the tests were conducted. The research was conducted in accordance with the Helsinki Declaration of 1975. Written informed consent was obtained from all participants. All data were anonymized to ensure confidentiality.

Data analysis

Descriptive statistics for continuous variables were presented as the mean ± SD for normally distributed data and median with interquartile ranges for non-normally distributed data. Normality was evaluated using multiple tests, including the Anderson-Darling, D'Agostino-Pearson, Shapiro-Wilk, and Kolmogorov-Smirnov tests. For group comparisons, the following statistical tests were employed: 1) the parametric unpaired Student's t-test for normally distributed variables; 2) the non-parametric Mann-Whitney U test for variables that violated normality assumptions; and 3) Z-tests for comparison of two population proportions. All inferential statistics were conducted at a 95% confidence interval. Statistical analyses were performed using Minitab® Statistical Software (State College, USA).

## Results

The mean age of the 37 adult control subjects, comprising both sexes, was 40.9 years. Among the 32 sex-matched patients, the mean age was 36.6 years in the LC-no group (n = 16) and 42.4 years in the LC-yes group (n = 16). The average BMI was 27.3 in the control group, 27.6 in the LC-no group, and 28.7 in the LC-yes group. The mean interval from acute SARS-CoV-2 infection to interview was 11.9 months (range: 6-24 months) in the LC-no group and 15.5 months (range: 7-29 months) in the LC-yes group.

During acute SARS-CoV-2 infection, the most frequently reported symptoms among patients included tiredness (24 cases, 75%), loss of taste (22 cases, 68.8%), fatigue (20 cases, 62.5%), loss of smell (19 cases, 59.4%), headache (18 cases, 56.3%), fever (15 cases, 46.9%), cough (14 cases, 43.8%), myalgia (14 cases, 43.8%), sore throat (12 cases, 37.5%), dyspnea (11 cases, 34.4%), rhinorrhea (9 cases, 28.1%), and diarrhea (8 cases, 25%). Notably, none of the patients reported phlegm. With the LC-yes group, 15 cases (93.7%) reported tiredness and/or fatigue compared to 10 cases (62.5%) in the LC-no group. Furthermore, 11 cases (68.8%) in the LC-yes group experienced six or more symptoms during the acute phase, whereas only 5 cases (31.3%) in the LC-no group reported similarly high symptom burden. A Z-test comparing these proportions yielded a p-value of 0.034, indicating a statistically significant difference (p < 0.05).

SARS-CoV-2 infection was confirmed by PCR test in 28 cases (87.5%) and by antibodies in 4 cases (12.5%). Regular daily medications were used in 8 cases (50%) of the control group, 4 cases (25%) of the LC-no group, and in 7 cases (43.7%) of the LC-yes group. However, no evidence was found to suggest that the use of these medications affected the ICC score [6].

**Figure 1:**
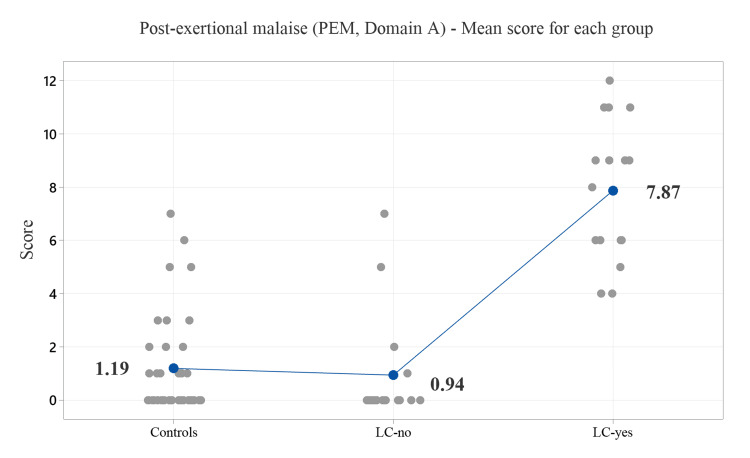
The plot displays the mean PEM scores for all groups (Domain A) Each of the five questions (as detailed in the text) was assigned a score: (0) no symptoms, (1) occasionally, (2) about half the time, (3) most of the time, and (4) constant symptoms. PEM: Post-exertional malaise

Table 2 shows the ICC scores for all groups [6]. ME/CFS was diagnosed in 4 cases (10.8%) of the control group (with a 3:1 female-to-male ratio), 1 case (6.3%) of the LC-no group (female), and in 3 cases (18.8%) of the LC-yes group (all male). The symptoms are listed in order of frequency, from most to least common. No significant differences were observed in the rates of ME/CFS diagnosis among the groups. Figure 1 displays the average symptom scores across the five PEM subcategories.

**Table 2:** Comparison of symptom prevalence among controls, LC-no, and LC-yes groups, showing significantly higher rates (p < 0.05) in LC-yes for key symptoms A comparison was made between the proportion of symptoms in control subjects and cases with SARS-CoV-2 infection who reported no lingering or new symptoms after six months (LC-no) and those with ongoing or new symptoms after six months (LC-yes). Symptom scores were evaluated using the ICC. Significant differences (p < 0.05) were found between the LC-yes group and both the LC-no group and controls for PEM; neurosensory, perceptual, or motor disturbances; neurocognitive impairments; sleep disturbances; pain; loss of thermoregulatory stability; flu-like symptoms; and increased susceptibility to viral infections with prolonged recovery periods. LC: Long COVID; ICC: International Consensus Criteria; PEM: Post-exertional malaise

Main symptoms	Domain	Control	LC-no	LC-yes	LC-yes/Control	LC-yes/LC-no
PEM	A	11 (29.7%)	4 (25%)	16 (100%)	< 0.00001	< 0.00001
Neurosensory, perceptual, or motor disturbance	B	7 (18.9%)	3 (18.7%)	16 (100%)	< 0.00001	< 0.00001
Neurocognitive impairments	B	11 (29.7%)	2 (12.5%)	15 (93.7%)	< 0.00001	< 0.00001
Sleep disturbance	B	10 (27%)	3 (18.7%)	12 (75%)	< 0.00112	< 0.00124
Pain	B	10 (27%)	1 (6.2%)	11 (68.7%)	< 0.00512	< 0.00028
Loss of thermoregulatory stability	D	8 (21.6%)	1 (6.2%)	10 (62.5%)	< 0.00362	< 0.00084
Gastrointestinal symptoms	C	7 (18.9%)	2 (12.5%)	6 (37.5%)	< 0.13622	< 0.10100
Flu-like symptoms	C	1 (2.7%)	0	4 (25%)	< 0.00714	< 0.03236
Susceptibility to viral infections with prolonged recovery periods	C	1 (2.7%)	0	4 (25%)	< 0.01078	< 0.03236
Cardiovascular symptoms	D	1 (2.7%)	3 (18.7%)	2 (12.5%)	< 0.12602	< 0.63122
Sensitivities to food, medications, odors, or chemicals	C	1 (2.7%)	1 (6.2%)	1 (6.2%)	< 0.44726	1.0
Intolerance of extreme temperatures	D	0	0	1 (6.2%)	< 0.12356	< 0.30772
Genitourinary symptoms	C	0	0	0	1.0	1.0
Respiratory symptoms	D	0	0	0	1.0	1.0

## Discussion

Main findings

PEM was present in all LC-yes cases (16/16, 100%) and 4 LC-no cases (25%). LC-yes individuals often reported persistent acute-phase symptoms, especially fatigue, and occasionally developed new post-acute symptoms such as PEM or sleep disturbances. ME/CFS was diagnosed in 4 controls (10.8%), 1 LC-no case (6.7%, female), and 3 LC-yes cases (18.8%, all male). Notably, ME/CFS typically demonstrates a 3:1 female-to-male ratio [9-11]. While this was reflected in the control group, this sex distribution was absent in the LC-yes group, suggesting potentially distinct pathophysiological mechanisms. Six or more acute symptoms were reported in 68.8% (11 cases) of the LC-yes group, compared to 31.3% (5 cases) of the LC-no group. This finding supports a potential association between greater acute symptomatology and increased risk of developing LC.

Long COVID and myalgic encephalomyelitis/chronic fatigue syndrome

ME/CFS and LC may present with similar symptoms, but there is no consensus on the percentage of LC cases that meet the diagnostic criteria for ME/CFS [7,9,12-14]. ME/CFS is diagnosed when symptoms meet the ICC criteria and cannot be attributed to other clinical or psychiatric conditions [6,7]. Reports on the likelihood of LC fulfilling the ME/CFS diagnostic criteria vary widely, with figures ranging from as low as 5% to as high as 56.8% of patients months after SARS-CoV-2 infection [4,13,15]. This wide variation could be due to outdated diagnostic criteria, such as the Fukuda criteria, which do not account for many central nervous system dysfunctions [8]. As a result, this can artificially inflate the number of LC cases diagnosed with ME/CFS [16]. Data analysis of 739 patients revealed only an 8.4% prevalence of ME/CFS among LC patients, with those infected during the Omicron phase showing a significantly lower prevalence of ME/CFS [17]. Similarly, Tokumasu et al. found that 17.9% of LC cases were diagnosed with ME/CFS, which closely aligns with our finding of 3 cases (18.8%) [14].

Long COVID

Regardless of the ME/CFS diagnosis, fatigue remains the most common symptom of LC, affecting 50% to 70% of patients, and it is likely of central origin [18-21]. This fatigue is often linked to “brain fog,” which involves memory problems, lack of mental clarity, poor concentration, and an inability to focus. Other common symptoms include cough, dyspnea, sleep disturbances, adjustment disorders, headache, anosmia/ageusia, difficulty concentrating, memory loss, confusion, arthralgia, and cognitive impairments [9,12,18,22-25]. In Latin America, the prevalence of LC has been estimated at 47.8%, with individuals reporting symptoms three months after SARS-CoV-2 infection [24]. In Brazil, a telephone-based survey in São Paulo, conducted among both hospitalized and non-hospitalized COVID-19 patients, estimated the prevalence of LC at 47.1% and 49.5%, respectively [26]. A Canadian study of 88 hospitalized patients found that 66.7% patients experienced fatigue three months after infection, decreasing to 59.5% after six months. The overall pooled prevalence of LC among hospitalized and non-hospitalized patients is 43%, with higher rates in hospitalized patients (54%) than in non-hospitalized individuals (34%). Additionally, women are more frequently affected than men (49% vs 37%) [27]. Notably, the development of LC does not seem to be strictly linked to the severity of the initial illness or the patient’s age [3,28]. Risk factors for LC include female sex, repeated infections, and more severe initial infection [5].

Fatigue

Although various muscle abnormalities have been proposed as potential causes of LC myalgia and fatigue, clinical inflammatory myopathy, characterized by limb-girdle weakness, dysphagia, and neck weakness, is rarely observed in LC cases. Since millions of people experience LC symptoms, it is challenging to explain these symptoms purely as a muscle pathology in any condition presenting with myalgia and fatigue. In a group of COVID-19 patients with mild to moderate symptoms, skeletal muscle injury, defined by elevated creatine kinase (CK) levels (>200 IU/L) and clinical scores, was found in 22.4% of cases [29]. These findings were mainly based on clinical presentation and serum CK levels, without using tools like needle electromyography, muscle biopsy, or muscle-specific antibodies for a more thorough assessment. This limitation makes it difficult to distinguish among muscle injury, acute post-viral syndromes, and peripheral neuropathy. It’s also important to recognize that both LC and ME/CFS patients may have sensory or autonomic small-fiber neuropathy, which can show up as impaired heat detection and increased tortuosity of small fibers in the central corneal subbasal plexus [30]. However, these features do not provide clear diagnostic criteria to distinguish between patients with LC and those with ME/CFS unrelated to LC. A recent study found normal values for isolated muscle fiber conduction velocity in situ and jitter parameters in the tibialis anterior muscle in cases of SARS-CoV-2 infection, with or without LC-related fatigue [21]. As shown in our earlier research, while jitter and muscle fiber conduction velocity tests revealed no dysfunction at the neuromuscular junction or within the muscle fibers, researchers suggest that fatigue may originate centrally, meaning the central nervous systsem might not be sending strong enough signals to sustain muscle performance [21].

Limitations

Several limitations should be acknowledged. First, the relatively small sample size may limit the generalizability of our findings. Second, although both control subjects and patients using medications were included, the proportions were comparable across groups, with the LC-yes group exhibiting a lower prevalence of medication than the controls. Third, this study did not assess the potential long-term effects of SARS-CoV-2 vaccination on symptom improvement, which remains a subject for future investigation, as vaccination may potentially reduce the duration or severity of LC symptoms.

## Conclusions

The presence of multiple symptoms (six or more) during the acute phase of SARS-CoV-2 infection appears to be associated with an increased risk of developing LC. In most cases, LC-related PEM endures from the acute infection phase, rather than emerging as a new symptom. The predominant manifestations of LC comprise PEM; neurosensory, perceptual, or motor disturbances; cognitive impairments; sleep disturbances; pain; loss of thermoregulatory stability; and flu-like symptoms, with marked differences compared to other groups. Within the LC-yes cohort, PEM was reported in all cases (16/16, 100%); however, a diagnosis of ME/CFS, as defined by the ICC, was established in only 3 cases (18.8%). These findings indicate that while LC shares substantial symptom overlap with ME/CFS, the latter may not adequately encompass the clinical presentation of most LC cases.

## References

[1] Zhu N, Zhang D, Wang W (2020). A novel coronavirus from patients with pneumonia in China, 2019. N Engl J Med.

[2] (2025). WHO COVID-19 dashboard. https://covid19.who.int/.

[3] Yong SJ (2021). Long COVID or post-COVID-19 syndrome: putative pathophysiology, risk factors, and treatments. Infect Dis (Lond).

[4] Nikolich JŽ, Rosen CJ (2023). Toward comprehensive care for long Covid. N Engl J Med.

[5] Ely EW, Brown LM, Fineberg HV (2024). Long Covid defined. N Engl J Med.

[6] Carruthers BM, van de Sande MI, De Meirleir KL (2011). Myalgic encephalomyelitis: International Consensus Criteria. J Intern Med.

[7] Twomey R, DeMars J, Franklin K, Culos-Reed SN, Weatherald J, Wrightson JG (2022). Chronic fatigue and postexertional malaise in people living with long COVID: an observational study. Phys Ther.

[8] Fukuda K, Straus SE, Hickie I, Sharpe MC, Dobbins JG, Komaroff A (1994). The chronic fatigue syndrome: a comprehensive approach to its definition and study. International Chronic Fatigue Syndrome Study Group. Ann Intern Med.

[9] Bateman L, Bested AC, Bonilla HF (2021). Myalgic encephalomyelitis/chronic fatigue syndrome: essentials of diagnosis and management. Mayo Clin Proc.

[10] Raveendran AV, Jayadevan R, Sashidharan S (2021). Long COVID: an overview. Diabetes Metab Syndr.

[11] Salari N, Khodayari Y, Hosseinian-Far A, Zarei H, Rasoulpoor S, Akbari H, Mohammadi M (2022). Global prevalence of chronic fatigue syndrome among long COVID-19 patients: a systematic review and meta-analysis. Biopsychosoc Med.

[12] Davis HE, Assaf GS, McCorkell L (2021). Characterizing long COVID in an international cohort: 7 months of symptoms and their impact. EClinicalMedicine.

[13] Kedor C, Freitag H, Meyer-Arndt L (2022). A prospective observational study of post-COVID-19 chronic fatigue syndrome following the first pandemic wave in Germany and biomarkers associated with symptom severity. Nat Commun.

[14] Tokumasu K, Honda H, Sunada N (2022). Clinical characteristics of myalgic encephalomyelitis/chronic fatigue syndrome (ME/CFS) diagnosed in patients with long COVID. Medicina (Kaunas).

[15] Phillips S, Williams MA (2021). Confronting our next national health disaster - long-haul Covid. N Engl J Med.

[16] Komaroff AL, Bateman L (2021). Will COVID-19 lead to myalgic encephalomyelitis/chronic fatigue syndrome?. Front Med (Lausanne).

[17] Morita S, Tokumasu K, Otsuka Y (2024). Phase-dependent trends in the prevalence of myalgic encephalomyelitis/chronic fatigue syndrome (ME/CFS) related to long COVID: a criteria-based retrospective study in Japan. PLoS One.

[18] Mahmud R, Rahman MM, Rassel MA, Monayem FB, Sayeed SK, Islam MS, Islam MM (2021). Post-COVID-19 syndrome among symptomatic COVID-19 patients: a prospective cohort study in a tertiary care center of Bangladesh. PLoS One.

[19] Malik P, Patel K, Pinto C, Jaiswal R, Tirupathi R, Pillai S, Patel U (2022). Post-acute COVID-19 syndrome (PCS) and health-related quality of life (HRQoL) - a systematic review and meta-analysis. J Med Virol.

[20] Baker AM, Maffitt NJ, Del Vecchio A, McKeating KM, Baker MR, Baker SN, Soteropoulos DS (2023). Neural dysregulation in post-COVID fatigue. Brain Commun.

[21] Kouyoumdjian JA, Yamamoto LA, Graca CR (2025). Jitter and muscle fiber conduction velocity in long COVID fatigue. Arq Neuropsiquiatr.

[22] Buttery S, Philip KE, Williams P (2021). Patient symptoms and experience following COVID-19: results from a UK-wide survey. BMJ Open Respir Res.

[23] Alkodaymi MS, Omrani OA, Ashraf N (2022). Prevalence of post-acute COVID-19 syndrome symptoms at different follow-up periods: a systematic review and meta-analysis. Clin Microbiol Infect.

[24] Angarita-Fonseca A, Torres-Castro R, Benavides-Cordoba V (2023). Exploring long COVID condition in Latin America: its impact on patients' activities and associated healthcare use. Front Med (Lausanne).

[25] Kisiel MA, Lee S, Malmquist S (2023). Clustering analysis identified three long COVID phenotypes and their association with general health status and working ability. J Clin Med.

[26] Malheiro DT, Bernardez-Pereira S, Parreira KC (2024). Prevalence, predictors, and patient-reported outcomes of long COVID in hospitalized and non-hospitalized patients from the city of São Paulo, Brazil. Front Public Health.

[27] Sharma SK, Mohan A, Upadhyay V (2024). Long COVID syndrome: an unfolding enigma. Indian J Med Res.

[28] Abrams RM, Zhou L, Shin SC (2023). Persistent post-COVID-19 neuromuscular symptoms. Muscle Nerve.

[29] Rajput SS, Aghoram R, Wadwekar V, Nanda N (2023). Skeletal muscle injury in COVID infection: frequency and patterns. Muscle Nerve.

[30] Seeck M, Tankisi H (2023). Clinical neurophysiological tests as objective measures for acute and long-term COVID-19. Clin Neurophysiol Pract.

